# Implementation and update of guideline-derived quality indicators for cervical cancer in gynecological cancer centers certified by the German Cancer Society (DKG)

**DOI:** 10.1007/s00432-023-05132-z

**Published:** 2023-07-15

**Authors:** Frederik A. Stuebs, Matthias W. Beckmann, Tanja Fehm, Christian Dannecker, Markus Follmann, Thomas Langer, Simone Wesselmann

**Affiliations:** 1 Department of Gynecology and Obstetrics, Erlangen University Hospital, Comprehensive Cancer Center Erlangen-European Metropolitan Area of Nuremberg (CCC ER-EMN), Friedrich-Alexander-Universität Erlangen-Nürnberg, Universitaetsstrasse 21–23, 91054 Erlangen, Germany; 2 Department of Gynecology and Obstetrics, University Hospital of Düsseldorf, Düsseldorf, Germany; 3 Department of Obstetrics and Gynecology, University, Hospital Augsburg, 86156 Augsburg, Germany; 4 German Cancer Society e.V., 14057 Berlin, Germany

**Keywords:** Cervical cancer, Quality indicator, Certified gynecological cancer centers, S3-guideline

## Abstract

**Purpose:**

In 2008, the first gynecological cancer centres were certified by the German Cancer Society (DKG). Guideline-based quality Indicators (QIs) are a core element of the certification process. These QI are defined to assess the quality of care within the centres and can serve to measure the implementation of guideline recommendation. This article aims to give an overview of the developing and updating process of guideline based-QIs for women with cervical cancer and presents the QI results from the certified gynaecological cancer centres.

**Methods:**

The QIs are derived in a multiple step review process and then implemented in the certification data sheet of the certified centres. The first set of QIs created in 2014 was revised in the update process of the S3-Guideline in 2020. QIs are based on strong recommendations of the evidence-based “Guideline for patients with Cervical Carcinoma” (registry-number: 032/033OL).

**Results:**

In total, there are nine guideline-based QIs for cervical cancer. Four QIs are part of the certification process. In the treatment year 2020, 3.522 cases of cervical cancer were treated in 169 centers. The target values for the four QIs were met in at least 95% of the certified centers. In the guideline update in 2020, a new QI was added to the set of QIs “Complete pathological report on conization findings” and the QI “Exenteration” was removed.

**Conclusions:**

QIs derived from strong recommendations of a guideline are an important tool to make essential parts of patient’s care measurable and enable the centers to draw consequences in process optimization. Over the years, the number of certified centers has grown, and the quality was improved. The certification systems is under constant revision to further improve patient’s care in the future, based on the results of the QI re-evaluation.

## Introduction

Cervical cancer is caused by a persistent infection with human papilloma virus (HPV) (Stuebs et al. 2021). Since the introduction of national screening programs in Germany in 1971, the incidence and mortality of cervical cancer have declined for three decades (Stuebs et al. 2022). However, despite advances in diagnosis and therapy, the incidence and mortality of cervical cancer have been stagnating at a low level in the past 15 years (Stuebs et al. 2022, 2019; Beckmann et al. 2021a, b; Fehm et al. 2021; Krebs in Deutschland für 2017/2018. Berlin: RKI 2023). In 2019, 4575 women in Germany were diagnosed with cervical cancer and 1597 died of the disease (Zentrum für Krebsregisterdaten im Robert Koch-Institut 2022). The 5-year relative overall survival is 65%, but strongly depends on the stage at first diagnosis.

In 2008, the German Cancer Society (DKG) together with the Germany Society for Gynecology and Obstetrics (Deutsche Gesellschaft für Gynäkologie und Geburtshilfe e. V. [DGGG]) initiated a certification system for gynecological cancer centres (Beckmann et al. 2014). In certified gynecological centres, patients are treated along the entire patient pathway in an interdisciplinary and multi-professional network. For certification, all disciplines must prove that they provide care for their patients based on the evidence-based guidelines and meet the qualitative and quantitative standards, which are summarised in a so-called catalogue of requirements and data sheet. The quality indicators derived from the guidelines are a central component of these standards (Kowalski et al. 2017). As of 31.03.2023, a total of 189 gynecological cancer centers are certified; of these, 17 centers are outside of Germany (Jahresbericht der deutschen Krebsgesellschaft (DKG) - Gynäkologische Krebszentren 2023). The main task of the DKG certification system is to ensure a high standard of quality in treating cancer patients in certified gynecological cancer centers (Rückher et al. 2022).

The guidelines are developed and revised under the supervision of the German Guideline Program in Oncology (GGPO) of the DKG, the German Cancer Aid, and the Association of the Scientific Medical Societies in Germany (AWMF) (Leitlinienprogramm Onkologie (Deutsche Krebsgesellschaft e.V., Stiftung Deutsche Krebshilfe, Arbeitsgemeinschaft der Wissenschaftlichen Medizinischen Fachgesellschaften (AWMF) e.V.) 2021). The funding was done by the German Cancer Aid (Project-Number: 70112702). The German S3-Guidelines are based on evidence derived from a systematic literature review including systematic reviews, meta-analyses and randomized controlled trials. The evidence is assessed by a representative interdisciplinary and interprofessional expert team covering relevant guideline topics, including patient representatives. A formal consensus-building process under the supervision of GGPO is mandatory (Langer and Follmann 2015). The development of recommendation-based QIs which address areas with improvement potential in the patient pathway is mandatory in developing and updating S3-Guideline process (Langer et al. 2017; Griesshammer et al. 2022).

**Fig. 1:**
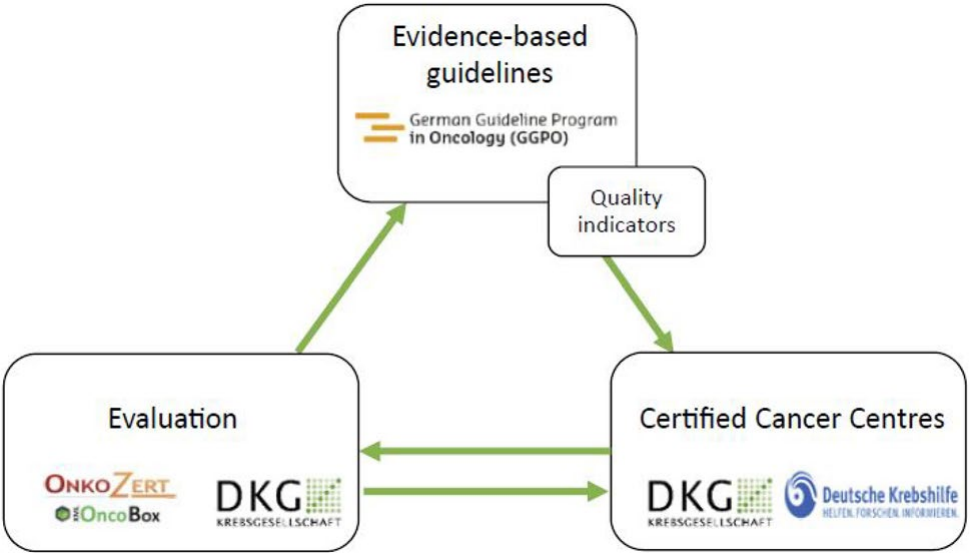
Quality cycle in oncology (Rückher et al. 2022)

The first S3-guideline for women with cervical cancer was published in 2014 and was updated in 2021 including the update of set of quality indicators. As a result of the German National Cancer Plan, the interaction between guideline and QI development, their application in certified centres and the use of the results for quality assurance and further development is summarised in a so-called quality cycle in oncology, which represents the interdisciplinary network (Fig. 1) (Rückher et al. 2022). This article presents the methodology of QI development in the context of the development of evidence based clinical guidelines, reports the results of these QIs from the certified cancer centers and the process of updating QIs (Rückher et al. 2022; Langendam et al. 2020; Nothacker et al. 2016).

## Methods

**Fig. 2:**
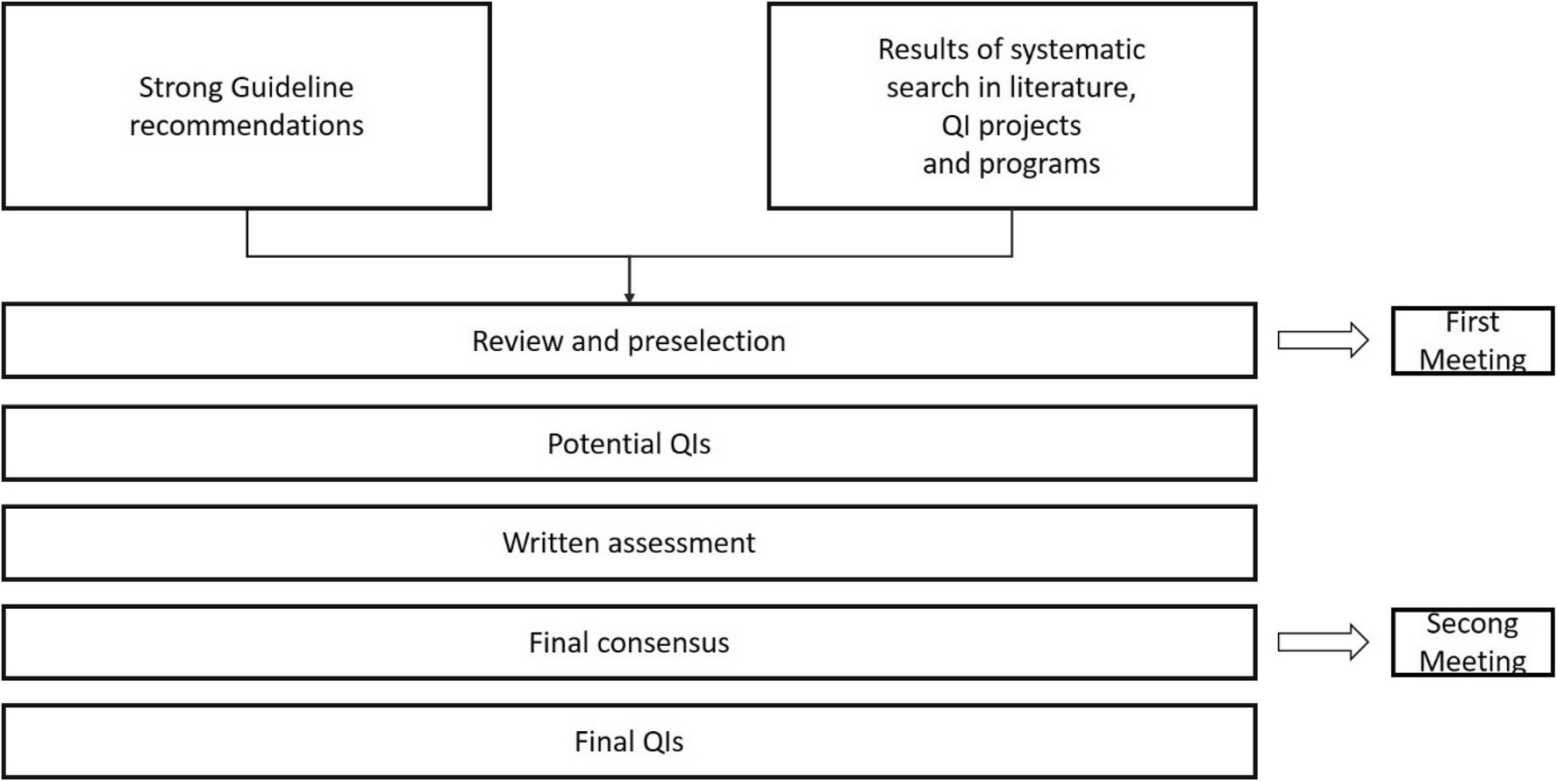
Flow chart QI development. *QI* quality indicator

A development process for QIs was set up by GGPO as shown in Fig. 2. The QI working group was composed of an interdisciplinary team of experts covering all relevant topics of the S3-guideline, including patient representatives, methodologists from GGPO, DKG certification, and AWMF and experts from the cancer registries (Leitlinienprogramm Onkologie (Deutsche Krebsgesellschaft, Deutsche Krebshilfe, AWMF) 2021; Leitlinienprogramm Onkologie (Deutsche Krebsgesellschaft, Deutsche Krebshilfe, AWMF) 2014; Follmann et al. 2020). Only strong recommendations of the S3-guideline with a grade of recommendation “A” according an intervention “should/should not” (German: “soll/soll nicht”) were eligible to be selected as QI candidate since it could be expected that the implementation of these recommendations will have a positive impact on the outcome of the patients in the addressed patient group (German Guideline Program in Oncology (German Cancer Society, German Cancer Aid, Association of the Scientific Medical Societies) 2023). The recommendations should be as specific as possible (German Guideline Program in Oncology (German Cancer Society, German Cancer Aid, Association of the Scientific Medical Societies) 2023).

Parallel a systematic literature review of English and German articles for already existing resp. additional national and international QIs was performed in the databases of PubMed and Cochrane. The search for the first QI-set (2014) did not have date limits. Studies reporting QIs for cervical cancer in all healthcare settings were included. The title and abstract of the extracted data were screened by two reviewers. Websites of known national and international institutions developing or publishing QI in oncology were screened manually, as well (Leitlinienprogramm Onkologie (Deutsche Krebsgesellschaft, Deutsche Krebshilfe, AWMF) 2021; Leitlinienprogramm Onkologie (Deutsche Krebsgesellschaft, Deutsche Krebshilfe, AWMF) 2014). The identified QIs were matched to the topics that are not (yet) covered by the strong recommendations. These identified QIs could give hints on further aspects for additional recommendations or modifications that have to be taken into account by the guideline panel (Rückher et al. 2022; Leitlinienprogramm Onkologie (Deutsche Krebsgesellschaft, Deutsche Krebshilfe, AWMF) 2021; Leitlinienprogramm Onkologie (Deutsche Krebsgesellschaft, Deutsche Krebshilfe, AWMF) 2014).

The QIs for the first version of the cervical cancer guideline were selected in a two-step process. At first, recommendations that could be considered for potential QIs were preselected in accordance with the GGPO methodology (Rückher et al. 2022; Follmann et al. 2020; German Guideline Program in Oncology (German Cancer Society, German Cancer Aid, Association of the Scientific Medical Societies) 2023). Criteria for exclusion of recommendations are shown in Table 1. The selected recommendations were transferred into potential QIs with clear numerator and denominator definitions. It was decided by the working group whether already existing QIs from the systematic search should be included in the set of potential QIs, if necessary, with an addition to the existing guideline recommendations. Then, in a second step, the working group members performed a written assessment of the potential QIs with a standardized sheet with four assessment criteria (Follmann et al. 2020; German Guideline Program in Oncology (German Cancer Society, German Cancer Aid, Association of the Scientific Medical Societies) 2023; ). Cancer registries and the certification system provide information about the availability of data. These information are necessary to assess the criterion “documentations effort” (see Table 2) (German Guideline Program in Oncology (German Cancer Society, German Cancer Aid, Association of the Scientific Medical Societies) 2023). A QI was accepted if the agreement was greater than or equal to 75% for each criterion mentioned in the assessment sheet. For more information on the selection process of QI, the methodology report of the GGPO is available on its webpage (https://www.leitlinienprogramm-onkologie.de/german-guideline-program-in-oncology/).

**Table 1:** Exclusion criteria for guideline recommendations

Number	Reason
1	Lack of feasibility
2	No potential for improving patient care through QI development
3	Lack of comprehensibility and/or great effort to collect data in proportion to benefit
4	Other reasons (possibility to enter free text)

*QI* quality indicator

**Table 2:** Criteria for the formal assessment of QI

Criteria for the formal assessment of QI
The quality indicator includes the potential for improving relevant patient outcomes
The indicator is clearly and unambiguously defined
The quality indicator is related to a supply aspect that can be influenced by the service provider
Are there any risks of incorrect control by the indicator that cannot be corrected?
The data is routinely documented by the service provider or an additional survey requiring a reasonable level of effort

For the update of an S3-Guideline, the QI-working group needed to be reconstituted. This working group checked whether the underlying recommendations of the existing QIs have changed and whether the existing QI needed to be adapted accordingly. It repeated the search for other national and international QIs covering the period since the first version (Leitlinienprogramm Onkologie (Deutsche Krebsgesellschaft, Deutsche Krebshilfe, AWMF) 2021) and carried out the selection and assessing process described above for newly defined or modified strong recommendations. On the basis of the results from the certified gynecological cancer centres, the experts of the QI-working group decided whether the existing QIs from the first version of the guideline should be maintained unchanged, modified or deleted resp. retired (Leitlinienprogramm Onkologie (Deutsche Krebsgesellschaft, Deutsche Krebshilfe, AWMF) 2021; German Guideline Program in Oncology (German Cancer Society, German Cancer Aid, Association of the Scientific Medical Societies) 2023).

The set of QIs from the first version of the guideline and the updated version were discussed in the meetings of the Certification Commission for Gynecological Cancer Centres. The members decided which QIs could be included in the data sheet, because they were applicable in the context of certified centres and which target values and plausibility limits should be applied to the QIs. The results of the implemented QIs were analysed and checked for plausibility and correctness in the annual on-site certification audits. The centres had to justify if and why they had not met the target values or plausibility limits of a QI. The results of all centres, including their explanations and the auditors' comments from the on-site audits, were summarised in the anonymised annual reports (Jahresbericht der deutschen Krebsgesellschaft (DKG) - Gynäkologische Krebszentren 2023).

With the introduction of the QIs, the certified gynecological cancer center’s outcomes as well as the practicability, plausibility and validity of each QI can be evaluated (German Guideline Program in Oncology (German Cancer Society, German Cancer Aid, Association of the Scientific Medical Societies) 2023).

In the following section, the set of cervical cancer QIs and the results of the implemented QIs will be presented.

## Results

In 2014, the working group selected the cervical cancer QI-candidates on the basis of 44 strong recommendations of the S3-Guideline. The search for already existing national and international QIs let to five further potential QIs, which were included in the selection process. The discussion in the QI working group led to the inclusion of a new specific objective to the guideline (recording the current care situation to avoid incorrect therapies in the future) and a new guideline recommendation (presentation in tumour conference) by the guideline group. Of the nine potential QIs that were assessed by the members of the QI working group, all were included in the final set of QIs. (see Table 3).

**Table 3:** Quality indicators (QIs) for cervical carcinoma defined by the working group QI

QI		
1	Presentation at the tumor conference (checked 2021)	Numerator: no. of patients presented at the tumor conference
		Denominator: patients with a first diagnosis, recurrence, or newly developed distant metastasis of cervical carcinoma
2	Details given in the pathology report at first diagnosis and tumor resection (checked 2021)	Numerator: no. of patients with pathology reports including details on:—histological type (WHO)—grading-evidence/absence of lymphatic or venous invasion (L and V status)—evidence/absence of perineural sheath infiltration (Pn status)—staging (pTNM and FIGO) in patients who have undergone conization, taking the conization findings into account—depth of invasion and extent in millimeters in pT1a1 and pT1a2—depth of invasion relative to the thickness of the cervical wall (metric or percentage) in radical hysterectomy-Three-dimensional tumor size in centimeters (starting from pT1b1)—minimum distance to the resection margins (in pT1b tumors, endocervical stroma)-R classification (UICC)
		Denominator: all patients with a first diagnosis of cervical carcinoma and tumor resection
3	Details in the pathology report with lymphadenectomy (checked 2021)	Numerator: no. of patients with pathology reports including details on:—no. of affected lymph nodes relative to removed lymph node Correlation with site of biopsy removal (pelvic/para-aortic)-Details of the largest extent of the largest lymph-node metastasis, in mm/cm—details of the absence/presence of capsular penetration by the lymph-node metastasis—details of isolated tumor cells or micrometastases
		Denominator: all patients with cervical carcinoma and lymphadenectomy
4	Cytological/histological lymph-node staging (checked 2021)	Numerator: no. of patients with cytological/histological lymph-node staging
		Denominator: patients with cervical carcinoma in FIGO stage ≥ IA2–IVA
5	Cisplatin-containing radiochemotherapy (checked 2021)	Numerator: no. of patients with cisplatin containing chemotherapy
		Denominator: all patients with a first diagnosis of cervical carcinoma and primary radiochemotherapy
6	Adjuvant radio(chemo)therapy (checked 2021)	Numerator: no. of patients with adjuvant radio(chemo)therapy
		Denominator: all patients with a first diagnosis of cervical carcinoma and radical hysterectomy
7	Histological confirmation (checked 2021)	Numerator: no. of patients with pretherapeutic histological confirmation
		Denominator: all patients with cervical carcinoma and treatment for a local recurrence
8	Diagnosis of spread in local recurrence (checked 2021)	Numerator: all patients with imaging diagnosis (CT of chest and abdomen) to exclude distant metastases
		Denominator: all patients with local recurrence of cervical carcinoma
9	Exenteration (deleted 2021)	Numerator: no. of patients with local R0 resection
		Denominator: all patients with cervical carcinoma and tumor recurrence and exenteration
10	Complete pathology report on conization findings (new 2021)	Numerator: all patients in the denominator with medical reports on:—type of lesion (CIN, AIS, SMILE)—location (endocervical, ectocervcal)—extent—in case of invasion, with details of size and lymphnode invasion, vascular invasion, and perineural sheath invasion—grading—status of resection margins (R status)
		Denominator: all patients with HSIL (CIN II/III), AIS, SMILE and/or cervical carcinoma who have undergone conization. Data on this indicator are to be collected by dysplasia units/services and gynecological cancer centers

All nine QIs were included in the data sheet for the gynecological cancer centres from 2015. However, in 2017, it was decided by the certification commission to limit the number of QI to the five most important QIs for each entity. This was necessary to prevent an overly demanding documentation burden, because all gynecological entities, e.g., ovarian cancer, endometrial cancer, cervical cancer, and vulvar cancer, are treated in the gynecological cancer centres, and thus, the associated, tumour-specific QIs also have to be documented. (Kurzprotokoll zur Sitzung der Zertifizierungskommission Gynäkologische Krebszentren 2017). In the same year, the absolute patient numbers for the numerators and denominators of the QIs were reported for the first time and not only the median and range as in the previous annual reports. The following five QIs have been included the data sheet for the certified centers (Kennzahlenauswertung 2022): “Presentation at the tumor conference”[QI1], “Details given in the pathology report at first diagnosis and tumor resection” [QI2], “Details in the pathology report with lymphadenectomy” [QI3] “Cytological/histological lymph-node staging” [QI4] and “Exenteration” [QI9] (Kennzahlenauswertung 2022). In the treatment year 2019, the QI “Exenteration” was excluded from data sheet due to the low number of exenterations (43) performed in only 23 of the 149 the certified centers in 2020 (see Table 4).

**Table 4:** QI over Time (the years refer to the treatment years)

Quality indicator		2020			2019			2018			2017		
		Results/cases	Median [range]	Centers meeting (TV)	Results/cases	Median [range]	Centers meeting (TV)	Results/cases	Median [range]	Centers meeting (TV)	Results/cases	Median [range]	Centers meeting (TV)
1	N	3426	16 [3–89]	100% (169/169)	3426	16 [5–80]	99.38% (161/162)	3053	17 [4–81]	100% (149/149)	2906	16 [4–74]	36.69% (51/139)
	D	3522	16 [3–89]		3511	16 [5–83]		3165	17 [4–81]		3040	17 [4–87]	
2	N	1492	7 [0–42]	95.83 (161/168)	1449	7 [1–33]	92.55% (149/161)	1175	6 [0–31]	99.33% (148/149)	1047	6 [0–34]	56.83% (79/139)
	D	1597	7 [1–45]		1590	7 [1–38]		1461	8 [2–36]		1429	8 [1–39]	
3	N	1215	5 [1–34]	100% (166/166)	1220	6 [1–31]	99.36% (155/156)	1137	5.5 [1–32]	100% (148/148)	1094	6 [0–38]	34.31% (47/137)
	D	1230	5 [1–36]		1248	6 [1–31]		1210	6 [1–32]		1207	6 [1–39]	
4	N	1495	7 [0–42]	98.82% (167/169)	1406	6 [0–36]	97.53% (158/162)	1346	6 [0–37]	99.33% (148/149)	1287	7 [1–39]	85.61% (119/139)
	D	1978	10 [1–47]		1949	9 [1–53]		1702	9 [1–50]			9 [2–46]	
9	N	/	/	/	/	/	/	36	1 [0–7]	88.46% (23/26)	30	1 [0–5]	16.67% (4/24)
	D	/	/	/	/	/	/	42	1 [1–8]		/	1 [1–7]	/
Total number of pt with cervical cancer treated in certified gynecological cancer centers		3.522	/	/	3.511		/	3.165		/	/		3.040

*TV* target value

As part of the update of the S3-Guideline, the set of QI was revised in 2020. 13 new strong recommendations were added in the update of the S3-Guidelinie. One new QI was derived from these recommendations and included in the final set of QIs: “Complete pathology report on conization findings” [Q10] (Leitlinienprogramm Onkologie (Deutsche Krebsgesellschaft, Deutsche Krebshilfe, AWMF) 2022). The QI “Exenteration” [Q9] was also excluded from the guideline set of QI’s. Thus, the final set of QIs in 2020 also consisted of 9 QIs (see Table 3).

The first gynecological cancer centers were certified in 2008. The number of gynecologic cancer centers has since then increased to 182, as of 31.12.2022 (Kennzahlenauswertung 2022). In the treatment year 2020, in total, 15.254 primary cases with the first diagnosis of a gynecological cancer were treated in certified cancers. Cervical cancer was the third most common cancer (primary cases) [*n* = 2.664 (17.46%)] after endometrial cancer [*n* = 4.753 (31.16%)] and ovarian cancer [*n* = 4.250 (27.86%)] (Kennzahlenauswertung 2022). The total number of primary cases treated in certified gynecologic cancer centers has increased from the treatment years 2015 (*n* = 11.587) to 2020 (*n* = 15.254). Of the 2.664 patients with cervical cancer, 2.587 were treated in centres in Germany. This represents 60% of the incident cervical cancer cases in Germany.

Since 2017, the results for five QIs are annually reported by the gynecological cancer centers and published in the annual reports (Jahresbericht der deutschen Krebsgesellschaft (DKG) - Gynäkologische Krebszentren 2023).

### Q1: Presentation at the tumor board

This QI comprises all women presented at an interdisciplinary tumor board with a first diagnosis, recurrence, or newly developed distant metastasis of cervical cancer. Since 2017, the proportion of women presented at the tumor board has been stable at a very high level (2017–2020: median 100%) In 2020, all centers met the target value of 80%. The minimum value steadily increased from 66.67% in 2017 to 80% in in 2020.

### Q2: Details in the pathology report in the case of initial diagnosis and tumor resection (checked 2021)

In recent years, the rate of pathology reports with detailed description of the tumor has increased from 73.27% in 2017 to 93.43% in 2020. In 2020, seven centers of 168 centers included in the annual report did not meet the target value of “≥ 80%”. The minimum value was 0% for the years 2017, 2018 and 2020. In 2019, the minimum value was 28.57%.

### QI3: Details in the pathology report in the case of lymphonodectomy

In 2020, all centers were above the target value (≥ 80%). In 2017 and 2018, the number of complete pathology reports was slightly lower with 90.64% and 93.97%, respectively. Since 2019, at least 98% of the centers were above the target value. The minimum value steadily increased from 0% in 2017 to 80% in in 2020.

### QI4: Cytological/histological lymph-node staging

Patients with cytological/histological lymph-node staging in patients with cervical cancer in FIGO stage ≥ IA2-IVA at least 98.82% of the gynecological cancer centers were within the plausibility limits (mandatory statement of reasons < 0.01%). In 2017, 85.61% of the centers were within the plausibility limits. The minimum values were 3.85% in 2017 and 0% in the other years.

### QI9: Exenteration

For the QI, data from treatment years 2017 and 2018 are available. In 2018, only in 26 centers, valid information for an exenteration was available. 123 centers did not perform an exenteration at all.

## Discussion

An important base of the certification process are recommendations of the evidence based guideline. The process of deriving QI including the selection and evaluation preceding its implementation in the certification process has been described above. The QIs are used to assess the degree of implementation of guideline recommendations in clinical practice and thus guideline adherence in the treatment of oncological patients.

The five quality indicators of the guidelines show very good results in the certified centers in the treatment years 2017–2020 resp. 2018 for QI 9 (see Table 4).

One of the goals of the German National Cancer Plan is the "intersectoral, integrated oncological care through interdisciplinary cooperation, for example in tumour boards, as well as intersectoral and interprofessional networking” (Health, F.M.o. 2023). Presenting all patients with cervical cancer in an interdisciplinary tumorboard for therapy planning has become standard of care in certified centers, which can be illustrated by the fact that 98% (3426 out of 3522 pat) of all eligible patients were presented 2020 in the tumor boards of the 169 centers who have been included in the annual report. In 2020, for the first time, all centers met the target value ≥ 80%. In 2019, only one center did not meet the plausibility limits, because two patients died before the tumor conference.

The pathology report for women with primary cervical cancer who undergo tumor resection needs to include the following histological details: histological type (WHO)—grading-evidence/absence of lymphatic or venous invasion (L and V status)—evidence/absence of perineural sheath infiltration (Pn status) (see Table 3). A complete pathology report is crucial for the treatment of cervical cancer patients. If three or more risk factors (e.g., L, V and Pn status) are identified, a primary radiochemotherapy is indicated (German Guideline Program in Oncology (German Cancer Society, German Cancer Aid, AWMF) 2022). It is, therefore, crucial for gynecologists to make sure that all risk factors are reported by the pathologists. This QI of the guideline cervical cancer continues to develop very well. Over the years most often information on the three-dimensional size, pN-status and minimal resection margins were missing. The centers sought discussions with the pathology departments to be able to submit complete pathology reports of findings in the future (Jahresbericht der deutschen Krebsgesellschaft (DKG) - Gynäkologische Krebszentren 2023).

For all patients with cervical cancer and lymphadenectomy, there should be a detailed pathology report including the following: number of affected lymph nodes relative to removed lymph node, correlation with site of biopsy removal (pelvic/para-aortic), details of the largest extent of the largest lymph-node metastasis (in mm/cm), details of the absence/presence of capsular penetration by the lymph-node metastasis and details of isolated tumor cells or micrometastases (German Guideline Program in Oncology (German Cancer Society, German Cancer Aid, AWMF) 2022). The results for the indicator improved over the years. In 2020 for the first time, all centers fulfilled the target value of 80% and 93.4% of all centers had a complete report for all surgical cases with lymphonodectomy. In 2019, all but one center fulfilled the 80% target. In that center, the information on the extent of the largest lymph node metastasis was missing for two patients. The specimens were re-examined and a quality cycle with the pathology department was organized to provide complete reports in the future.

The surgical staging or interventional diagnosis plays a key role in defining the histological tumor stage, which is crucial for planning the correct treatment strategy. Conventional imagings such as CT, MRI and PET–CT are not sufficiently sensitive or specific in the detection of lymph-node metastases (Lande et al. 2007; Altgassen et al. 2008; Selman et al. 2008). A meta-analysis, including 72 studies and 5042 women, compared the surgical staging using the sentinel-node method in cervical cancer with various imaging methods (CT; MRI, PET–CT) (Selman et al. 2008). Using the sentinel-node method, a sensitivity of 91.4% were reported in comparison with 74.7% (for PET-CT), 55.5% (for MRI), and 57.5% (for CT) and a specificity of 100% in comparison with 97.6% (for PET-CT), 93.2% (for MRI), and 92.3% (for CT) (Selman et al. 2008). This indicates the superiority of surgical staging over imaging techniques. Small metastases in particular often remain undetected on conventional imaging ( German Guideline Program in Oncology (German Cancer Society, German Cancer Aid, AWMF) 2022). In 2020, only two centers have not performed a surgical staging for their patients (previous year four centers). The two centres had three and one patient in the denominator, and referred to patients with best supportive care and Hb-effective bleeding, respectively, which was taken as a reason for rapid surgery. In the previous years, a common reason stated by the centers not to perform a surgical staging were the comorbidities of the patients, age of the patients or suspicious lymphnodes in conventional imaging. Fortunately, this QI has steadily improved over the years and the recommendations of the S3-Guideline have been gradually implemented in clinical routine to improve the treatment of women with cervical cancer.

In 2020, the QI exenteration was no longer included in the data sheets, because in the previous year, 123 centers did not carry out an exenteration. 19 of the remaining 26 centers achieved an R0 resection rate of 100%. The three centers with a 0% rate only performed an exenteration on one patient. Due to the good implementation and at the same time low number of exenterations performed, the quality indicator has been deleted from the data sheet.

The number of certified gynecological cancer centers and hence the number of women treated because of cervical cancer within these centers steadily increased since the introduction of the certification system. This is all the more relevant, because a large retrospective cohort study using statutory health insurance data has shown that there is a significant survival advantage when patients with cervical carcinoma are treated in certified centres (HR 0.84, CI 0.76–0.92) (WiZen - Wirksamkeit der Versorgung in onkologischen Zentren - Ergebnisbericht.pdf 2022).

There are limitations in the process of setting up QIs and implementing them in data sheets for certified centers. Quality of life of patients are not considered in setting up QIs. The whole content of guideline cannot be covered by QIs. QIs are evaluated and discussed in the onsite audits one calendar year after the treatment year of the patients. Therefore, the structure and healthcare process might be different in the local centers and thus the framework conditions for the QI results. The introduction of digital medicine might be a chance: If the QIs are measured and reflected in parallel with patient’s treatment, deviations from the quality objectives of a QI can be reacted to in a timely manner. Important for successful implementation of QIs is the acceptance of clinicians. The expenditure for documenting the QI needs to be practical. Considering these aspects, only a small set of QIs is transferred to data sheets. The number of QIs was further reduced by certification commission to five QIs per entity. The QI “Exenteration” was also excluded as the number of exenterations performed was too small for a valid evaluation. The majority of centers did not perform exenterations at all. This shows that QIs need to be reevaluated continuously.

The implementation and evaluation of guideline-derived QIs for women with cervical cancer can help to measure and influence the high-quality patient’s care positively. As crucial part of the quality cycle in oncology (see Fig. 1) QIs have proven to be a valuable tool to improve the quality in diagnosis and treatment over time. (Rückher et al. 2022; Butea-Bocu et al. 2021; Beckmann et al. 2011; Haj et al. 2017; Kreienberg et al. 2018; Trautmann et al. 2018).

In addition, the results of the QI can be used in the certification process to identify areas with potential for improvement. Centers that miss the target values have the opportunity to justify the deviation and discuss this during the audits and adequate measures can be agreed between the centre and the auditors, which are suitable for improving the QI results (Rückher et al. 2022). The effectiveness of these measures can then be reviewed in the audit of the following year. Thus, a stable and effective process for quality improvement based on guideline QI is implemented in the certified centres.

Especially, for cancer types not that common such as cervical cancer, systematic QI implementation and evaluation may help to generate broader databases and thus broadens knowledge to improve care and treatment of the affected women.

## Data availability statement

The datasets generated during and/or analysed during the current study are available from the corresponding author on reasonable request.
